# ASO Author Reflections: Real-World Results of the KEYNOTE-522 Regimen to Guide Surgical Decision Making

**DOI:** 10.1245/s10434-025-16931-8

**Published:** 2025-01-29

**Authors:** Casey Connors, Julie E. Lang

**Affiliations:** 1 Department of Breast Surgery, Cleveland Clinic, Lerner Research Institute, Cleveland, OH USA; 2 Department of Cancer Biology, Cleveland Clinic, Lerner Research Institute, Cleveland, OH USA

## Past

Triple-negative breast cancer (TNBC), which is notorious for its aggressive tumor biology and overall poorer prognosis, accounts for approximately 15% of all breast cancer diagnoses.^1–3^ The KEYNOTE-522 trial introduced immunotherapy into the neoadjuvant setting for patients with stage II/III TNBC, resulting in improved pathologic complete response (PCR) rates and event-free and overall survival.^4^ Surgical candidacy for breast-conservation therapy (BCT) can increase following neoadjuvant systemic therapy (NAT) but is often time contingent upon the degree of clinical response. In our study, we sought to evaluate whether patients undergoing NAT using the KEYNOTE-522 regimen in a real-world setting had improved pCR that correlated with an increased rate of breast conservation compared with patients who underwent NAT using the prior standard-of-care regimen (control group).^5^ Additionally, we set out to evaluate BCT eligibility, effect on axillary surgery, identification of clinical characteristics to help predict pathologic response to NAT, identification of factors that influence surgery type, mean tumor reduction, and immune-related adverse events (irAEs).

## Present

Our findings provide real-world outcomes of the KEYNOTE-522 regimen along with its impact on pathologic response and surgical procedure. Our results demonstrated a pCR rate of 59% following NAT using the KEYNOTE-522 regimen compared with 33% using the prior standard of care regimen. Despite this significant improvement in pCR following chemoimmunotherapy, there was no significant increase in BCT rates or BCT candidacy compared with that of the control group. There was however a significant correlation with increased pCR and surgical de-escalation in the axilla, with a resulting 14% decrease in the rate of axillary lymph node dissection following the KEYNOTE-522 regimen. Interestingly, we found that patients harboring a BRCA1 mutation had a 75% pCR rate with standard-of-care chemotherapy, which was not significantly different following the KEYNOTE-522 regimen. This is important when considering the irAEs of immunotherapy, which was noted to be 35% in our patient population.

## Future

Our study provides insight into treatment using the KEYNOTE-522 regimen outside of a clinical trial. The results demonstrate a significant increase in pCR, which did not have an impact on the rate of breast conservation in this patient population. The significant reduction in axillary nodal disease following the KEYNOTE-522 regimen will help to decrease the number of patients who undergo unnecessary axillary lymph node dissections. Our results provide awareness into the anticipated response to NAT using the KEYNOTE-522 regimen, which can help guide surgical discussions in the pre-NAT and presurgical settings. Further studies are required to determine whether there is any benefit of NAT with immunotherapy for patients with BRCA1 mutations, who were found to have a superior pCR with prior standard of care. Should comparable results be found, this subset of patients could be considered for an individualized approach, sparing them from immunotherapy and its potential irAEs. For all patients, a better understanding of the long-term effects of immunotherapy will help the multidisciplinary team better advise patients on the risks and benefits of chemoimmunotherapy.
